# Improvements in sleep, weight, and eating-disorder symptoms following off-label leptin treatment in an adolescent with chronic anorexia nervosa and insomnia: a case report

**DOI:** 10.1186/s40337-026-01748-x

**Published:** 2026-08-27

**Authors:** Alexander Dück, Hang Zhu, Judith Melinat, Monika Kruzer, Christoph Berger, Michael Kölch, Martin Reinhardt, Hannes Brehme, Annelie Zimmermann, Linda Frintrop, Olaf Reis

**Affiliations:** 1 Department of Child and Adolescent Psychiatry and Neurology, Rostock University Medical Center, 18147 Rostock, Germany; 2 Institute of Anatomy, Rostock University Medical Center, Rostock, Germany; 3 German Center for Child and Adolescent Health (DZKJ), Partner Site Greifswald/Rostock, Rostock, Germany

**Keywords:** Anorexia nervosa, Leptin, Sleep, Circadian rhythm, Motor activity, Starvation, Weight

## Abstract

We report off-label administration of human recombinant leptin (metreleptin) over a 12-day period in a 17-year-old female adolescent with chronic anorexia nervosa (AN) and clinically significant insomnia. Two observation windows are distinguished throughout: a short-term window between day 1 (pre) and day 12 (post) of dosing, and a longer-term window between day 12 and the two-month follow-up (d + 60), capturing both changes temporally associated with leptin administration and the course of continued weight gain after the end of dosing. Over the short-term window the clinical course comprised a modest weight gain (BMI 15.4 → 16.2 kg/m²), a marked reduction in eating-disorder cognitions and compulsive exercise, attenuation of starvation-associated motor hyperactivity, and changes in objective sleep architecture (more N3 sleep, less wake after sleep onset). In this window, broader cognitive-affective and depressive symptom dimensions remained largely unchanged, and subjective insomnia did not yet improve. Over the longer-term window the course comprised continued substantial weight gain (BMI 18.3 kg/m² at d + 60), a marked drop in subjective insomnia, partial normalisation of circadian rhythm parameters in nonparametric analysis, and a transient increase in broader cognitive-affective symptoms. As a single retrospective case observation, the findings are hypothesis-generating and cannot be disentangled from concurrent weight gain and inpatient treatment.

## Abstract (plain language summary)

Anorexia nervosa is a serious illness in which a person eats too little and loses a dangerous amount of weight. It is often very hard to treat, and many people with the illness also have problems with sleep. Scientists think that the hormone leptin, which the body makes in fat tissue, may play a part. People who have lost a lot of weight have very little leptin, and low leptin levels may be linked both to the restless, driven behaviour seen in the illness and to poor sleep.

In this report we describe the treatment of one 17-year-old girl who had been seriously ill with anorexia nervosa for a long time and also had difficulty sleeping. As an experimental treatment, and with the agreement of the patient and her parents, she was treated with metreleptin (a manufactured form of leptin) as a daily injection for 12 days, alongside her usual hospital care. We followed her closely during the 12 days and again about two months later, measuring her weight, her eating-disorder symptoms, her physical restlessness and her sleep, including with overnight sleep recordings and a movement sensor worn on the wrist.

During the 12 days of treatment she gained some weight, her eating-disorder thoughts and her urge to exercise lessened, she became less physically restless, and the deep, restful stage of her sleep increased. Her own sense of having insomnia did not improve straight away, but by the two-month follow-up it had clearly improved, and she had continued to gain weight. Some other symptoms, such as low mood, briefly became more noticeable again as she gained weight. The treatment was generally well tolerated, with no side effects reported.

Because this is a single case and the patient was also receiving normal treatment and gaining weight at the same time, we cannot prove that leptin caused these improvements. The findings should be seen as a starting point for further research rather than as proof that the treatment works. Carefully designed studies in larger groups are needed to find out whether leptin can genuinely help sleep and other symptoms in people with anorexia nervosa.

## Background

Anorexia nervosa (AN) is characterised by a triad of core features: low body weight, an intense fear of weight gain, and a marked disturbance in body image [59]. The somatic, psychological, and behavioural manifestations of the disorder are closely linked to the consequences of starvation that arise from sustained energy restriction [3, 17, 63, 39, 65].

### AN and leptin

Leptin, an adipocyte-derived hormone, plays a central role in the regulation of appetite, body weight, and glucose homeostasis [2]. The reduction in fat mass that defines AN is accompanied by a marked decrease in circulating leptin [11, 15, 31, 32]. Hypoleptinemia is therefore a key endocrine feature of AN and is considered a primary signal driving somatic and behavioural adaptations to starvation [33]. Clinically meaningful manifestations of starvation—hyper-motor activity, depressed mood, cognitive inflexibility, persistent fear of weight gain, intrusive thoughts about food, and sleep problems—may be initiated or exacerbated by hypoleptinemia [11, 34, 45].

In contrast to anorexia nervosa, experimental semi-starvation in individuals without an eating disorder reduces spontaneous physical activity [39]. The paradoxical hyperactivity of AN thus reflects a failure of the normal energy-conserving down-regulation rather than a universal effect of starvation [14, 51]. Notably, elevated physical activity can antedate substantial weight loss in a subset of patients and may represent a premorbid, trait-like feature [18, 19, 42].

Animal models of starvation-induced AN show that chronic food restriction leads to marked hyperactivity and circadian disruption associated with hypoleptinemia. In mice, starvation disrupts circadian rhythmicity, increases motor activity, alters hypothalamic clock-gene expression, and reduces glial cell density in the suprachiasmatic nucleus; these effects are largely reversible with refeeding [64]. In rats, continuous leptin administration prevents and reverses starvation-induced hyperactivity, identifying leptin deficiency as a key biological driver of starvation-related hyperactivity [22]. In patients with AN, higher leptin levels following weight gain are associated with reduced subjective motor restlessness [22]. This starvation-induced hyperactivity—especially the rise in locomotion preceding scheduled feeding—has been interpreted as displaced food-seeking (foraging) behaviour, i.e. an evolutionarily conserved drive to seek food during energy deficit [28, 55], although reward-related and thermoregulatory interpretations have also been proposed.

### AN and sleep

Genetic analyses indicate a bidirectional association between AN and a morningness chronotype, and a link between genetic liability for insomnia and AN, while no consistent associations are seen with sleep duration or daytime sleepiness [61]. Across age groups, poorer subjective sleep quality in AN is associated with greater illness severity and impaired quality of life, independently of BMI and negative emotional symptoms [7, 44]. Subjective sleep disturbances in eating disorders are further associated with greater depressive symptoms and higher eating-disorder severity [20].

These associations also hold for objective sleep measures. Actigraphy in AN shows more disturbed and irregular sleep patterns than in healthy controls, with more nocturnal awakenings and greater variability in sleep timing despite similar sleep duration [41]. Polysomnography in adolescents with restricting-type AN shows a marked reduction in slow-wave sleep and delta activity, indicating impaired sleep depth, that is linked to severity of starvation rather than to illness duration [48]. Across psychiatric disorders, sleep continuity disturbances represent a transdiagnostic feature, with disorder-specific alterations in sleep depth, sleep continuity, and NREM/REM architecture [5]. Accordingly, in AN polysomnographic and subjective data consistently show persistent impairments in sleep continuity and NREM architecture compared with healthy controls [1], which are not consistently normalised by weight restoration alone [12].

### Leptin and sleep

Experimental sleep restriction in humans reduces leptin levels and disrupts metabolic-endocrine regulation, demonstrating that sleep is a key regulator of leptin and energy homeostasis [56]. Local leptin administration in rodents promotes sleep by increasing NREM and REM sleep, enhancing slow-wave activity, and suppressing orexin-mediated arousal through direct action on hypothalamic sleep-wake nuclei. Mechanistically, leptin inhibits wake- and hunger-promoting AgRP neurons while activating POMC neurons, thereby counteracting starvation-induced hyperarousal and stabilising sleep-wake architecture [26, 49] .

### AN, leptin and sleep

In a controlled longitudinal polysomnography study by [43], AN was characterised by reduced slow-wave sleep, increased light sleep, and markedly decreased IGF-1 and leptin levels; higher IGF-1 and leptin concentrations were associated with longer and deeper sleep, and partial normalisation of sleep architecture and EEG power occurred after limited weight gain [43].

Recent publications have demonstrated that hypothesis-driven off-label treatment with metreleptin in patients with AN is associated with notable psychological benefits. Metreleptin administration was followed by a rapid reduction in hyperactivity, with objective reductions in motor activity and improved behavioural control prior to weight restoration [4]. In these case reports, sleep-related outcomes were limited to subjective self-report; only one case used a visual analogue scale, and none included objective sleep measurements [4, 27, 45]. The present case report aims to close this gap by combining subjective and objective measures of sleep quality during leptin-based treatment of AN, and by separating short-term effects of dosing from longer-term effects accompanying continued weight gain.

## Case presentation

### Pre-treatment evaluation

A 17-year-old female adolescent was admitted for inpatient treatment due to acute refusal of food and fluid that had persisted for several days. At referral, she reported a weight loss of 6 kg over the preceding month. Owing to dietary restriction and excessive physical activity, she was in a life-threatening state with a body weight of 38.2 kg and a body mass index (BMI) of 14.2 kg/m² (< 1st percentile). “Significantly low body weight” is the primary criterion (Criterion A) for the diagnosis of AN in the DSM-5. AN had been diagnosed several years earlier; since then she had received continuous treatment, including several stays in specialised eating-disorder units.

She showed a preoccupation with food and body weight, an inability to abandon a calorie-restricted diet, inner tension with episodes of non-suicidal self-injury, diminished concentration, increased nocturnal wakefulness, and depressed mood. At first, she regularly required supplemental nutrition via a feeding tube. Over time, this was offered as a supplement on days when she was unable to eat a full meal, and eventually became unnecessary even before the metreleptin treatment trial began. With a diet plan providing up to 2,500 kcal, her weight gain eventually leveled off. She showed limited insight into her condition and low motivation for further treatment. Her serum leptin level was very low at 1.3 ng/ml. According to published case series, average leptin levels at the time of referral are generally below 2 ng/ml [10, 23, 31].

From the day of admission the patient reported difficulties initiating and maintaining sleep, with prolonged nocturnal wakefulness and early morning awakening, present for a long time. The symptoms were experienced as persistently distressing but had not been addressed in previous treatments. Medical history revealed no organic cause. Criteria for an insomnia disorder were met. All clinical (blood pressure, heart rate, temperature) and laboratory parameters (blood cell counts, liver and kidney values, electrolytes) were within the normal range, and clinical parameters were monitored closely throughout treatment.

The decision to attempt an off-label trial of metreleptin was made against the background of an unusually long and severe course of illness with persistent failure to gain weight. AN had been diagnosed several years earlier, and despite continuous treatment over many months — including repeated inpatient stays in specialised eating-disorder units and, during the present admission, supplemental tube feeding in addition to a structured meal plan — weight remained critically low and had again plateaued. Given this chronic and treatment-refractory course, off-label metreleptin treatment was considered on an individual-case basis, drawing on the emerging published experience in AN, after standard nutritional and psychotherapeutic measures had not achieved sustained weight gain.

Both the patient and her parents provided informed consent for off-label treatment with metreleptin, having been thoroughly briefed on the available clinical experience with metreleptin in AN, including all known adverse effects and the FDA-mandated risk-management programme. Adherence to a regimen of three main meals (07:00, 11:30, 18:30) and three snacks (09:30, 15:30, 20:00) per day was emphasised to mitigate the risk of hypoglycaemia.

### Psychometric assessment

*Subjective AN symptoms.* German versions of the Child Eating Disorder Examination Questionnaire [13, 36], Eating Disorder Inventory-2 [58], Beck Depression Inventory-II [9], and Compulsive Exercise Test [52, 57] were completed at the start (d1, pre) and end (d12, post) of dosing, and again two months after cessation of dosing (d + 60, follow-up; [35]).

CHEDE-Q evaluates eating-disorder psychopathology in children and adolescents in four domains (Restraint; Eating Concern; Shape Concern; Weight Concern) and provides a global score (range 0–6; clinical cut-off ≥ 2.8; community mean ≈ 1.5; [46]). The pre-treatment global score of 4.7 indicated pronounced psychopathology across all domains, well above the clinical cut-off (see Table 1). EDI-2 captures broader cognitive and personality-related characteristics (e.g., drive for thinness, body dissatisfaction, ineffectiveness, perfectionism, interoceptive awareness, maturity fears). The EDI-2 is norm-referenced and has no single validated total cut-off [24]; the pre-treatment score lay in the range typically observed in clinically established AN (see Table 1). BDI-II assesses severity of depressive symptoms (0–13 minimal, 14–19 mild, 20–28 moderate, 29–63 severe; [8]). The pre-treatment score of 43 indicated severe depressive symptomatology (see Table 1). CET measures compulsive or problematic physical activity (e.g., exercise for weight/figure control, affect regulation, rigidity, guilt avoidance). On the standard CET metric (sum of the five subscale means; range 0–25), the pre-treatment total was 12.8, below the cut-off of 15 for clinically relevant compulsive exercise [57]; compulsive-exercise features were therefore in the upper subclinical range and decreased further during dosing (see Table 1).

During the 12 days of dosing the patient additionally completed a 20-item visual analogue scale (VAS; range 1–10) used by [45] and adapted in line with [29, 29, 45]. The VAS was completed twice daily (08:00 and 19:00). Items included hunger, repetitive thoughts of food, fear of weight gain, drive for activity, inner tension, feelings of fullness, nausea, perceptions of feeling fat, urge to self-harm, depressed mood, tiredness, interest in social interaction, mental narrowing, exhaustion, urge to binge, urge to vomit, dominated by the eating disorder, compulsive thinking, motivation to overcome the eating disorder, and suicidal thoughts. As patient-reported ratings, these twice-daily VAS data - together with the self-report questionnaires (ISI, CHEDE-Q, BDI-II) - provide a structured, indirect representation of the patient\u2019s own perspective on her symptoms and the treatment, albeit in quantitative rather than narrative form.

### Sleep assessment

Subjective insomnia was assessed with the German Insomnia Severity Index [21, 25] at d1, d12 and d + 60 as part of our clinical routine. Actigraphic recording (MotionWatch) was carried out throughout the inpatient phase as an adjunct to psychotherapeutic treatment, providing a means of monitoring motor activity. Retrospectively, two complete seven-day windows available before and during dosing were analysed using Cosinor analysis [16], and non-parametric circadian rhythm analysis (NPCRA) [62] was applied to two-day weekend windows recorded before, during, and after dosing. Mobile polysomnography (PSG) was performed in the patient’s room before dosing (d1) on differential-diagnostic grounds, given the clinically significant sleep disturbance. A second PSG was obtained for progress monitoring (d12), as the patient continued to report frequent early-morning awakenings despite weight gain under metreleptin treatment.

### Treatment

As mentioned earlier, the decision to pursue an off-label treatment attempt with metreleptin was prompted by the patient’s persistent failure to achieve meaningful weight gain despite a protracted treatment course, including repeated inpatient care. Metreleptin was administered subcutaneously into the thigh once daily at 08:30. The dose was titrated for safety from 3 mg (d1 + 2) to 6 mg (d3-6) to 9 mg (d7-12). As no effective dose has been established for anorexia nervosa, dosing followed a physiological-substitution rationale and was oriented on the weight-based scheme approved for generalised lipodystrophy (Myalept prescribing information) and on prior off-label case series in AN, in which metreleptin was titrated for safety up to approximately 9–11 mg/day [4, 27, 45]. Given the patient’s low body weight, the target was to raise the markedly reduced endogenous leptin toward a physiologically appropriate level rather than to reach a pharmacologically defined dose. Across 12 days of dosing and seven blood-glucose checks per day, blood glucose dropped below 5 mmol/L (90 mg/dL) on four occasions on four different days (lowest 4.6 mmol/L / 83 mg/dL), without symptoms of hypoglycaemia. No abnormalities were seen in repeated checks of blood pressure, heart rate, and temperature. The patient reported no side effects during dosing or at follow-up.

## Results

The following sections are structured according to two observation windows. To reduce the risk of scope creep inherent to a data-rich single case, we designate the primary focus of this report as the subjective and objective characterisation of sleep and circadian rest–activity organisation during and after metreleptin administration (polysomnography; actigraphy-based Cosinor and non-parametric circadian rhythm analysis; Insomnia Severity Index). Changes in body weight and in eating-disorder, depressive and compulsive-exercise symptomatology are reported as secondary, contextualising observations. This distinction is exploratory and was defined post hoc, in keeping with the single-case, non-pre-specified design. First, the short-term window between day 1 (pre) and day 12 (post) of dosing covers the clinical course while the patient was still underweight and receiving metreleptin. Second, the longer-term window between day 12 (post) and the two-month follow-up (d + 60) covers the course after cessation of dosing, during continued weight gain. All measurements reported here were obtained as part of routine clinical care and were clinically indicated; they constitute a retrospective documentation of the clinical course rather than pre-specified study endpoints. Within each window, findings are reported first for weight and symptoms of AN (including cognitions and visual-analogue ratings) and then for sleep quality and sleep disorder (subjective followed by objective measures). For the sake of clarity, the figures display the short- and longer-term windows together.

### Short-term window (days 1–12)

#### Weight and symptoms of AN

**Fig. 1:**
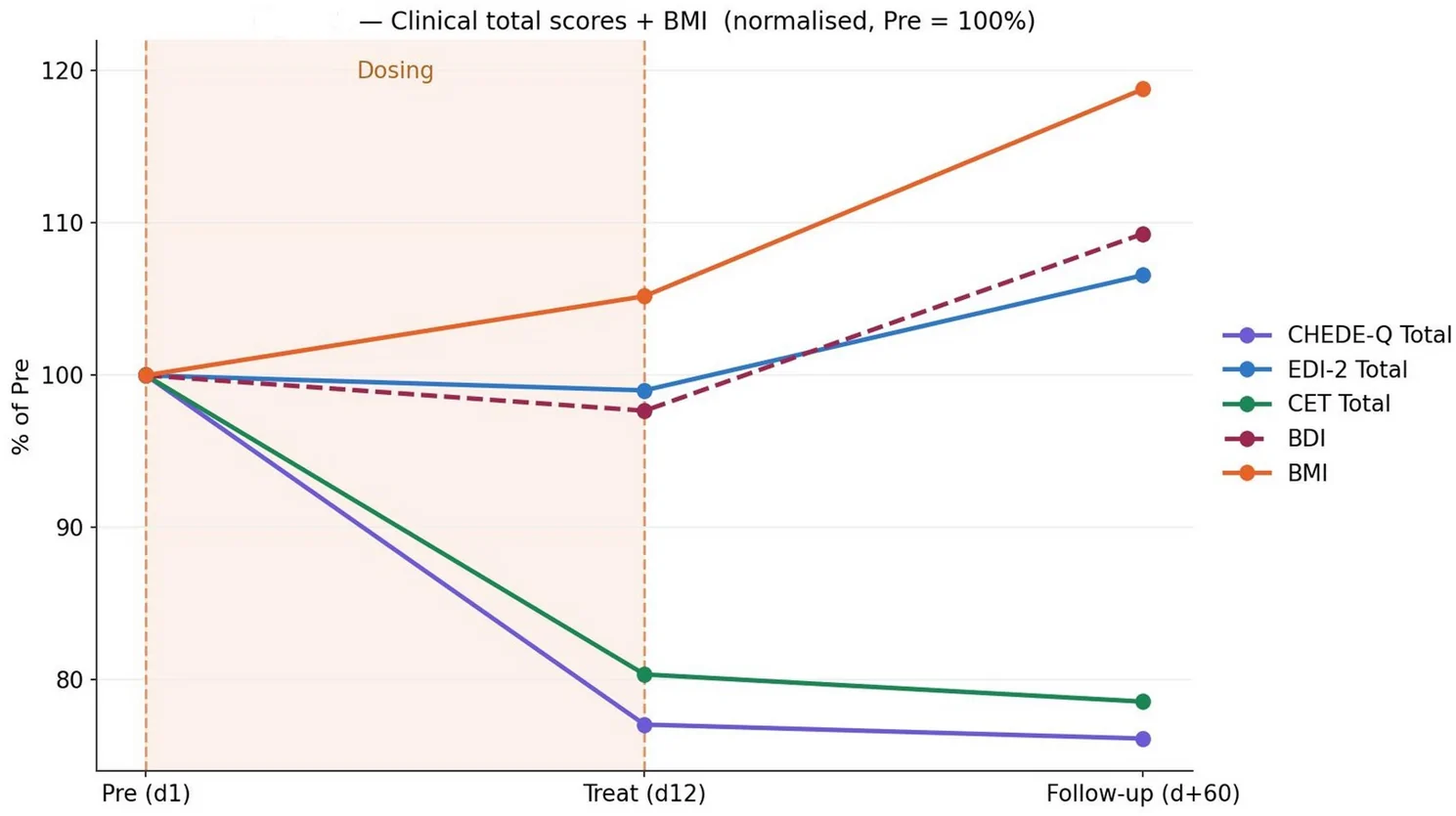
Normalised trajectories (Pre = 100%) of clinical total scores and BMI across the three assessment points (Pre/d1, Treat/d12, Follow-up/d + 60). Vertical dashed lines mark the boundaries of the dosing period. CHEDE-Q, Children’s Eating Disorder Examination Questionnaire; CET, Compulsive Exercise Test; EDI-2, Eating Disorder Inventory-2; BDI, Beck Depression Inventory; BMI, body mass index

Body weight and BMI (clinical measurement) increased during dosing (see Fig. 1). During the 12-day dosing period the patient gained 1.95 kg, with body weight rising from 41.55 kg (BMI 15.4 kg/m²; <1st percentile) on the day before first dosing to 43.50 kg (BMI 16.2 kg/m²; >1st percentile) on the last day of dosing, an increase of + 5%. During this period, feeding via a feeding tube was not necessary. Laboratory and clinical parameters remained within normal limits. Serum leptin had risen to 4.30 ng/mL, adjusted for BMI, Tanner stage and sex [6].

Alongside BMI, eating-disorder cognitions and behaviour, compulsive exercise, broader cognitive-affective characteristics and depressive symptoms were assessed at the beginning (d1) and end (d12) of dosing (see also Table 1).

**Table 1:** Self-rated psychological data for the patient prior to dosing (Pre, d1), at the end of metreleptin treatment (Treat, d12), and two months after cessation of dosing (Follow-up, d + 60)

	Pre (d1)	Treat (d12)	Follow-up (d + 60)
*CHEDE-Q*			
Total score	109	84	83
Restraint	24	19	19
Eating Concern	20	14	12
Weight Concern	25	16	17
Shape Concern	40	35	35
EDI-2 total score	304	301	324
BDI	43	42	47
*CET*			
Total score	56	45	44
Avoidance and rule-driven behaviour	13	12	8
Weight control exercise	12	10	12
Mood improvement	12	9	10
Lack of exercise enjoyment	12	8	8
Exercise rigidity	7	6	6

CHEDE-Q and CET are shown as raw summed scores. On the standard metrics, the EDE-Q global score (0–6; cut-off ≥ 2.8) was 4.7/3.5/3.5 and the CET total (0–25; cut-off ≥ 15) was 12.8/10.0/10.1 at d1/d12/d + 60; the EDI-2 is norm-referenced without a total cut-off

Over the dosing window the total scores diverged. Eating-disorder cognitions and behaviour (CHEDE-Q, − 23%) and compulsive exercise (CET, − 20%) both decreased markedly, with all subscales contributing in the same direction, whereas broader cognitive-affective characteristics (EDI-2, − 1%) and depressive symptoms (BDI-II, − 2%, remaining in the severe range) were essentially unchanged. Subscale-level values are given in Table 1.

Thus, the symptom dimensions most closely tied to starvation biology (CHEDE-Q and CET) decreased substantially during dosing, whereas the broader cognitive-affective and depressive dimensions (EDI-2 and BDI-II) remained essentially unchanged, despite the modest rise in BMI.

**Fig. 2:**
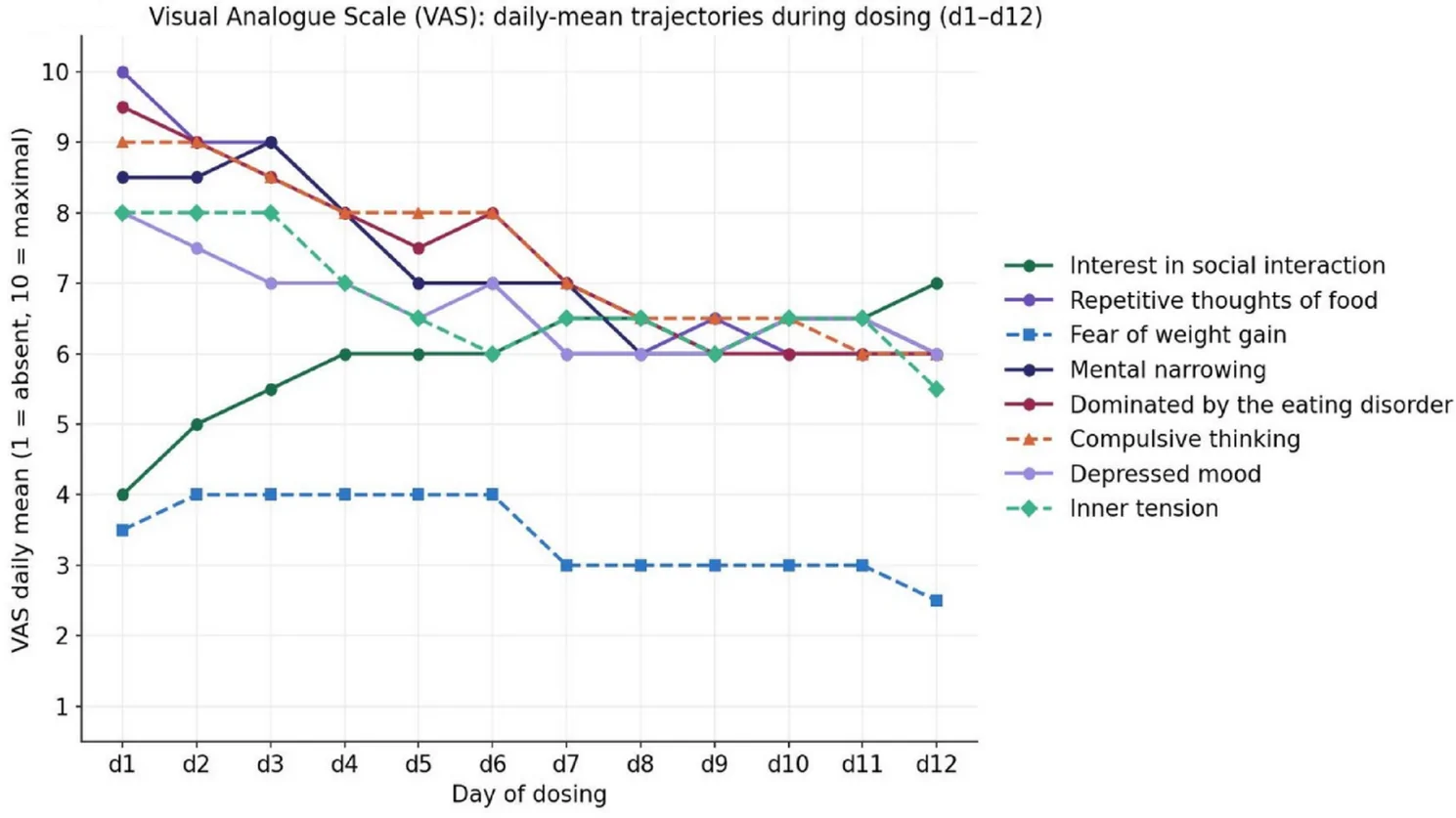
Visual Analogue Scale (VAS) ratings of selected items across the dosing period (days 1–12), scale range 1 (absence) to 10 (maximal intensity). Items were rated twice daily (08:00 and 19:00); each marker represents the daily mean for that item

Starvation-associated cognitions, affect and behaviour (visual analogue scale, VAS) were rated twice daily across the dosing window; daily mean trajectories for days 1–12 are shown in Fig. 2. Eight items showed a pronounced or progressive course. Seven decreased from d1 to d12: repetitive thoughts of food (Δ − 4.0), being dominated by the eating disorder (Δ − 3.5), compulsive thinking (Δ − 3.0), mental narrowing and inner tension (each Δ − 2.5), depressed mood (Δ − 2.0) and fear of weight gain (Δ − 1.0), while interest in social interaction increased (Δ + 3.0), compatible with progressive social re-engagement. Taken together, these trajectories describe a coherent attenuation of starvation-associated cognitive-affective arousal over the dosing window; item-level trajectories are shown in Fig. 2.

### Symptoms of sleep quality and sleep disorder

**Fig. 3:**
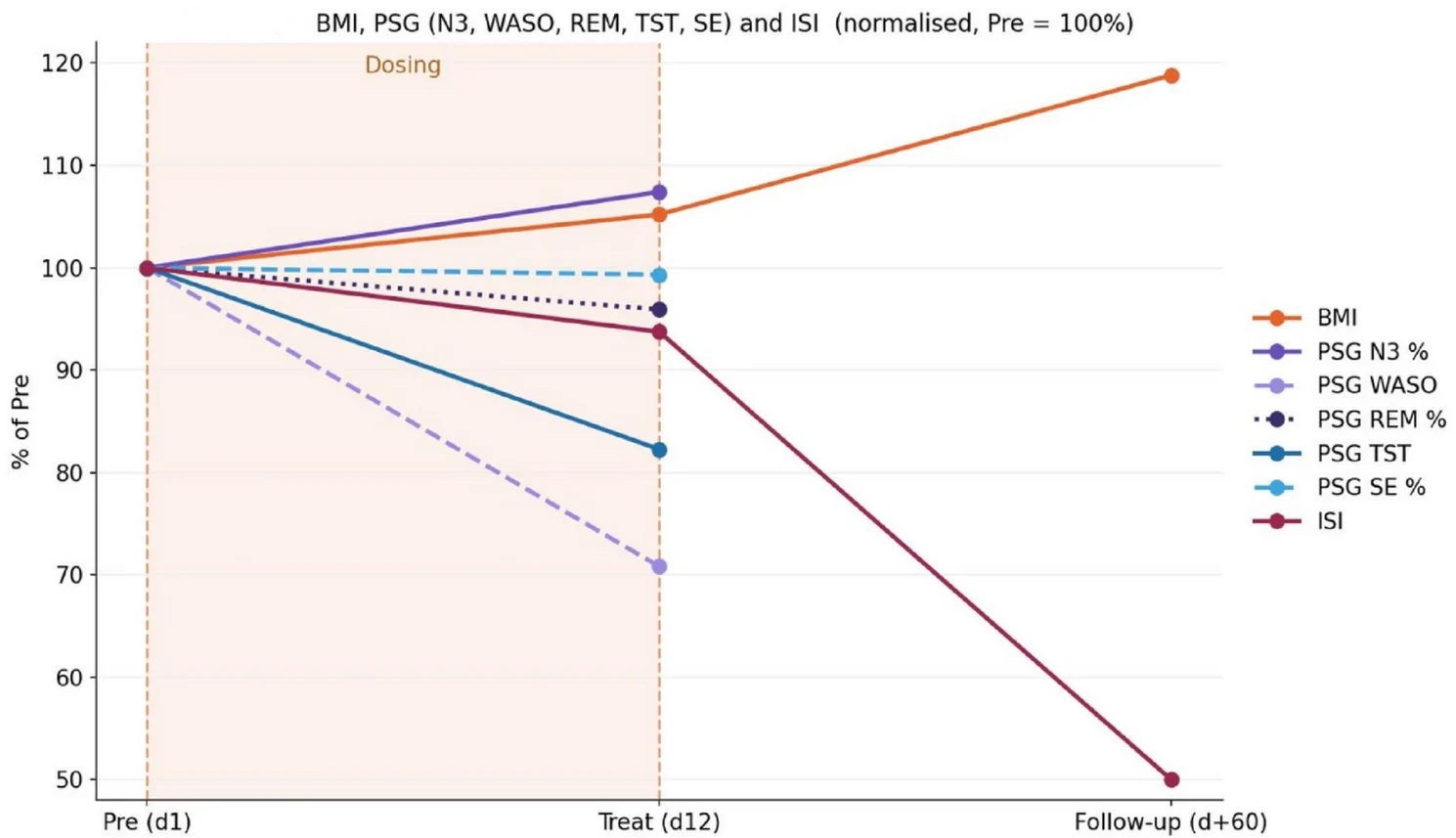
Normalised trajectories of BMI, polysomnographic sleep architecture and ISI across the three assessment points. BMI, body mass index; PSG, polysomnography; N3, slow-wave sleep; WASO, wake after sleep onset; REM, rapid eye movement sleep; TST, total sleep time; SE, sleep efficiency; ISI, Insomnia Severity Index; d, day

*Subjective insomnia (ISI)* remained essentially stable from d1 to d12, with the total score staying within the moderate insomnia range (see Fig. 3; Table 2).

**Table 2:** Insomnia Severity Index (ISI) total scores and severity classification at the three measurement points

Time point	Pre (d1)	Treat (d12)	Follow-up (d + 60)
Total score	16	15	8
Insomnia severity	moderate	moderate	subthreshold

Objective sleep architecture (polysomnography, PSG) was recorded twice (d1 and d12), so all PSG changes fall into the short-term window. Figure 3 shows the normalised trajectories of BMI, PSG parameters and ISI; Table 3 provides the absolute values.

**Table 3:** Polysomnographic parameters at d1 (PSG pre) and d12 (PSG post), with comparison against age-adjusted normative ranges [53] and direction of within-subject change

	PSG pre	Norm	PSG post	Norm	Change
Lights off [time]	21:00		22:00		
Lights on [time]	05:02		04:39		
Recording time [min]	542.6		501.2		↓
TIB [min]	482.1		399.2		↓
SPT [min]	472.6		384.7		↓
TST [min]	436.6	=	359.2	−	↓
SE [%]	90.6	=	90.0	=	↓
WASO [min]	36.0	+	25.5	=	↓
Sleep onset [min]	9.5	=	14.5	=	↑
REM latency [min]	76.5	=	108.0	=	↑
N1 [%]	6.4	=	6.8	=	↑
N2 [%]	37.0	=	34.2	−	↓
N3 [%]	41.9	+	45.0	+	↑
REM [%]	14.7	=	14.1	=	↓
Avg. HR during sleep [bpm]	63	=	64	=	↑
AHI	1.1	=	1.2	=	↑
Limb movement index [n/h]	4.3	=	4.7	=	↑
Arousal index	4.5	=	6.2	=	↑

= within age norm; + above age norm; − below age norm; ↑ increased after treatment; ↓ decreased after treatment. TIB (time in bed); SPT (sleep period time); TST (total sleep time); SE (sleep efficiency); WASO (wake after sleep onset); REM (rapid eye movement); N1, N2, N3 sleep stages; AHI (apnoea-hypopnoea index); % proportion of TST

*Total sleep time (TST)* decreased by −18% from d1 to d12; pre-treatment TST was within the age-adjusted normative range [53] and fell below norm at d12. *Wake after sleep onset (WASO)* decreased by − 29%, shifting from abov norm into the normative range. *Slow-wave sleep proportion (N3%)* increased by + 7%,with both values aove the age norm. *Sleep efficiency (SE)* remained virtually unchanged, within norm at both time points. *REM proportion (REM %)* decreased slightly by − 4%, within norm at bot time points. Sleep onset latency and REM latency both rose while remaining within norm; N1% rose slightly (withn norm) and N2% decreased, falling elow norm (see Table 3). Lights-off occurred later (21:00 → 22:00) and lights-on earlier (05:02 → 04:39), resulting in a shorter time in bed and recording time. Cardiorespiratory parameters were unremarkable across both nights (apnoea–hypopnoea index, average heart rate, limb movement index and arousal index all within age norm). Both nights showed early morning awakening (05:02 and 04:39); a long awake phase between midnight and 01:00 documented in the first night was absent in the second.

In summary, the short-term PSG course, recorded over the dosing window, was characterised by deeper but shorter sleep, with consolidated nocturnal continuity (lower WASO, higher N3%) at the cost of total sleep duration, while subjective insomnia (ISI) had not yet changed.

**Fig. 4:**
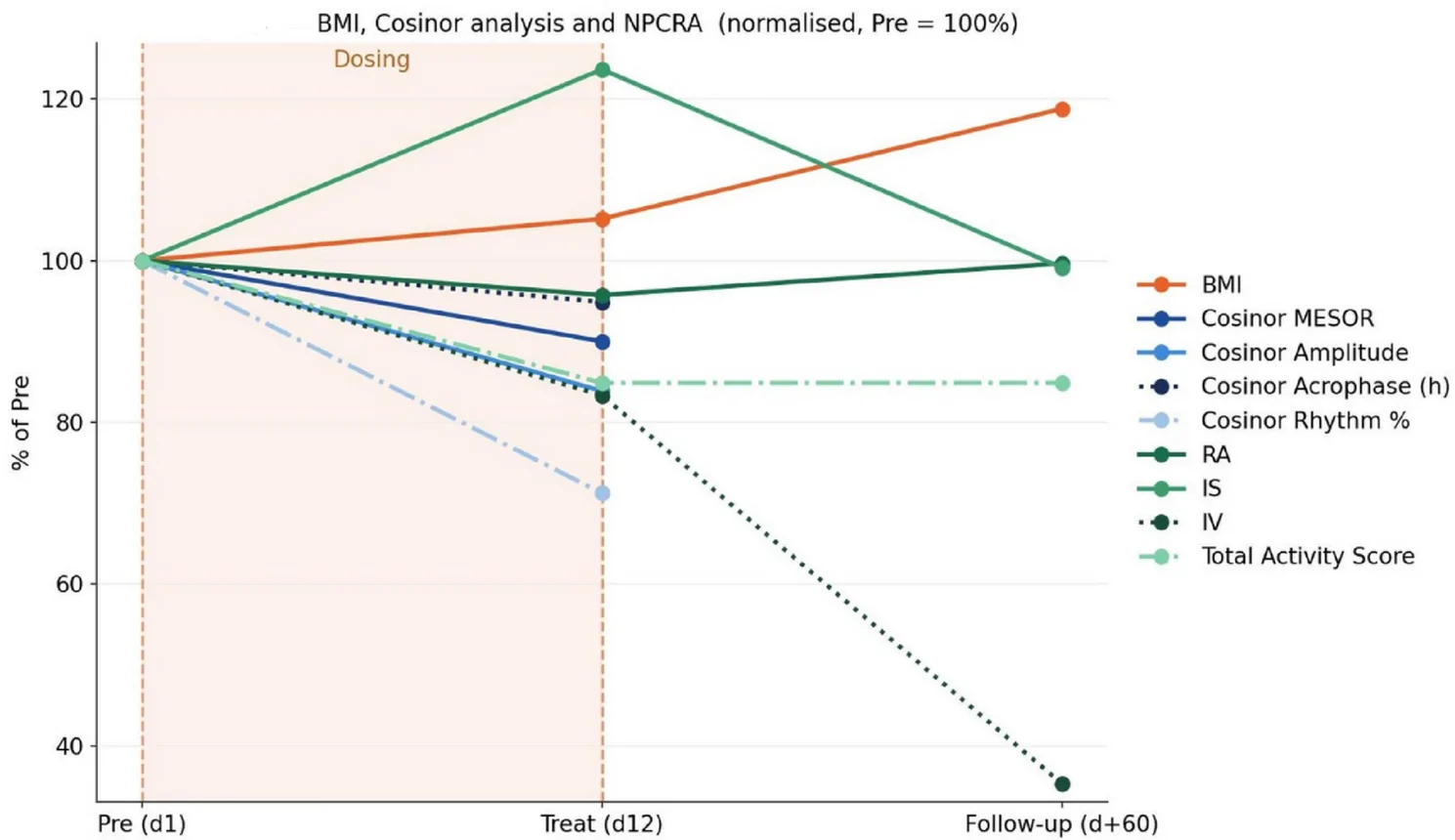
Normalised trajectories of BMI, Cosinor parameters and non-parametric circadian rhythm analysis (NPCRA) parameters across the three assessment points. BMI, body mass index; MESOR, midline statistic of rhythm; IV, intradaily variability; IS, interdaily stability; RA, relative amplitude; d, day

Objective motor activity and circadian organisation (actigraphy, Cosinor analysis) could be modelled only on the two complete seven-day windows preceding and during dosing; the post-dosing weekend recording was too short. All Cosinor changes therefore fall into the short-term window (Fig. 4; Table 4).

**Table 4:** Cosinor analysis of actigraphic activity data over the seven-day windows preceding dosing (Pre) and during dosing (Treat)

Parameter	Pre (avg.)	Treat (avg.)
MESOR	40.1854	36.1716
Amplitude	36.5038	30.6180
Acrophase (h)	14.33	13.60
Rhythm %	0.73	0.52

Only two complete seven-day windows could be analysed; the post-dosing weekend recording was too short for a full Cosinor model

MESOR (rhythm-adjusted mean activity) decreased by − 10%. Amplitude decreased by − 16%. Acrophase shifted earlier (14:20 → 13:36; see Table 4). Rhythm percent (variance explained by the 24-hour sine fit) decreased by − 29%

The pattern indicates reduced overall motor activity, a flatter daytime activity peak, an earlier peak time and a poorer fit to a sinusoidal model, i.e. a less rigidly over-driven daytime activity profile.

Objective within- and between-day activity regularity (actigraphy, non-parametric circadian rhythm analysis, NPCRA) was computed for two-day weekend windows before, during and after dosing (Fig. 4; Table 5). In the short-term window (d1 → d12), intra-daily variability (IV) decreased by − 17%, indicating less fragmented within-day activity; interdaily stability (IS) rose; relative amplitude (RA) decreased slightly; and the total activity score decreased by − 15%.

**Table 5:** Non-parametric circadian rhythm analysis (NPCRA; RA, IS, IV) and total activity score derived from MotionWatch recordings, comparing the two-day weekend windows before, during, and after dosing

Parameter	Pre (avg.)	Treat (avg.)	Post (avg.)
RA (Relative amplitude)	0.965	0.924	0.962
IS (Interdaily stability)	0.570	0.705	0.565
IV (Intra-daily variability)	0.102	0.085	0.036
Total activity score	2.559	2.172	2.172

### Longer-term changes (d12 → d + 60)

Longer-term changes concern only a portion of the short-term parameters. PSG and Cosinor analyses were available for the short-term window only and are therefore not reported again here.

### Weight and symptoms of AN

Body weight and BMI (clinical measurement) continued to rise from d12 to d + 60, reaching 18.3 kg/m² (+ 13% above d12 and + 19% above d1; Fig. 1). The trajectories of the self-rated total scores diverged.

*Eating-disorder cognitions and behaviour (CHEDE-Q)* remained essentially stable (− 1%). Among the subscales, Weight Concern rose slightly, Eating Concern continued to fall, and the remaining domains were unchanged.

*Compulsive exercise (CET)* changed only marginally (− 2%), with continued decrease in Avoidance and rule-driven behaviour but a return of Weight control exercise to its pre-treatment level.

*Broader cognitive-affective characteristics (EDI-2)* rose by + 8% from d12 to d + 60, exceeding the pre-treatment level.

*Depressive symptoms (BDI-II)* rose by + 12% from d12 to d + 60, also exceeding the pre-treatment level.

Thus, the dimensions captured by CHEDE-Q and CET — which had improved during dosing (markedly for the eating-disorder cognitions) — were maintained at the new lower level, whereas the dimensions captured by EDI-2 and BDI-II rose during the period of further weight gain (Fig. 1).

*Treatment satisfaction (TSQM*,* Version 1.4;* [60])*)* increased over the same period: the patient’s response to the item on satisfaction with the medication’s effectiveness in symptom management rose from 15 during the treatment phase to 17 at the follow-up visit.

### Symptoms of sleep quality and sleep disorder

*Subjective insomnia (ISI)* dropped substantially (a 7-point reduction representing a clinically meaningful improvement; [47]) from d12 to d + 60, from 15 (moderate) to 8 (subthreshold; Fig. 3; see Table 2), with the largest reductions in the items pertaining to sleep maintenance and early morning awakening. Subjective improvement in insomnia therefore occurred predominantly after the end of dosing, parallel to continued weight gain.

*Objective within- and between-day activity regularity (actigraphy*,* NPCRA)* continued to change after the end of dosing (d12 → post; Fig. 4; Table 5). IV continued to decrease (cumulative − 65% vs. d1); IS returned to its pre-treatment value; RA recovered toward its pre-treatment level; and the total activity score remained at the post-treatment level. Across both windows the day-night contrast represented by RA was preserved, while within-day fragmentation (IV) showed a sustained, progressive improvement; the transient increase in IS during dosing did not persist.

## Discussion

To our knowledge, the present case is the first in which changes in objective parameters — represented by PSG and actigraphic measures — are documented during off-label leptin treatment of an adolescent with AN, alongside subjective sleep, body and psychological changes. The observed pattern lends itself to a structured interpretation along the two observation windows defined above: a short-term window (d1 → d12), during which changes were temporally associated with metreleptin administration in a still-underweight state, and a longer-term window (d12 → d + 60), during which the course was dominated by continued weight gain after the end of dosing. As this is a single retrospective case observation, the changes described cannot be disentangled from concurrent treatment and weight gain, and the interpretations offered below are hypothesis-generating rather than confirmatory.

### Short-term window (d1 → d12)

The changes observed during the dosing window occurred while the patient was still underweight and receiving metreleptin. They were paralleled by only a modest concurrent increase in BMI. The short-term changes are therefore temporally associated with metreleptin administration and compatible with a leptin-related contribution, although they cannot be disentangled from the concurrent, if modest, weight gain. Because the density of clinical documentation was far greater in this window — daily self-ratings, two polysomnographic nights and continuous actigraphy — the short-term window also forms the empirical core of this case and is discussed here in the greatest detail.

### Weight and symptoms of AN

The 1.95 kg gain across 12 days is consistent with previous off-label metreleptin reports in AN, in which rapid weight gain was observed during dosing alongside improvements in eating-disorder symptoms [4, 27, 45]. Continued weight gain across the longer-term window is compatible with the possibility that early changes associated with leptin replacement persisted beyond the dosing period, although this cannot be separated from the continued inpatient treatment. Whether early leptin levels predict longer-term weight maintenance remains controversial [38, 40, 54]. It should be noted that the patient concurrently received treatment as usual, including supplemental tube feeding, a structured meal plan, and medication at a stable dosage. The weight gain can therefore not be attributed to metreleptin alone.

The self-rated symptom dimensions showed a clear dissociation during dosing (Fig. 1). The two dimensions most tightly coupled to starvation biology improved, most markedly the eating-disorder cognitions, while the two dimensions reflecting the broader and more enduring psychopathology of AN remained essentially unchanged.

Acute eating-disorder cognitions and behaviour — dietary restriction together with weight- and shape-related concerns (assessed with the CHEDE-Q) — decreased substantially within the dosing window. In parallel, compulsive exercise — the drive to be physically active and the use of exercise to regulate weight and affect (assessed with the CET) — also decreased, from an already subclinical baseline (CET total below the cut-off of 15; see Results). Both dimensions describe the restriction–hyperactivity phenotype that is characteristic of the starved state and that is closely mirrored by the rodent phenotypes of starvation-induced hyperactivity and altered feeding behaviour [22, 37, 64]. Their rapid attenuation during dosing, against the background of only a modest rise in BMI, is therefore compatible with a leptin-related contribution to these specific symptom dimensions of AN, while remaining temporally confounded with early weight gain.

By contrast, the broader cognitive and personality-related features of AN—such as ineffectiveness, perfectionism and interoceptive disturbance (assessed with the EDI-2) — and depressive symptoms (assessed with the BDI-II) did not change meaningfully during dosing. These dimensions capture longer-standing, partly trait-like aspects of the disorder that, based on the evidence reviewed in the Introduction, improve more slowly and may even transiently intensify during early weight restoration [12, 43]. Their stability across the 12-day window is in line with this expectation. The dissociation between these two sets of dimensions is one of the central observations of this case.

The twice-daily self-ratings (VAS) provided a fine-grained view of the cognitive, affective and behavioural symptoms of the starved state across the dosing window (Fig. 2). Seven dimensions attenuated progressively: intrusive food-related preoccupation, fear of weight gain, cognitive constriction (mental narrowing), the sense of being dominated by the eating disorder, compulsive thinking, depressed mood and inner tension; in parallel, interest in social interaction increased, reflecting progressive social re-engagement. Taken together, these trajectories describe a coherent reduction of starvation-driven cognitive-affective arousal. Because they were rated by the patient herself, the VAS trajectories also constitute an indirect, structured account of her subjective experience over the dosing window; in the absence of a separate first-person narrative, they - alongside the self-report questionnaires - are the principal means by which the patient´s own perspective enters this report. Their rapid attenuation under metreleptin parallels the rapid symptom improvements reported in earlier off-label cases [29, 45] and aligns with the conceptualisation of hypoleptinemia as a primary signal driving these symptoms [33, 34]. A leptin-related central-nervous contribution to these dimensions would be compatible with experimental evidence that leptin counteracts starvation-induced hyperarousal at the hypothalamic level [26, 49].

### Symptoms of sleep quality and sleep disorder

Perceived sleep quality—the patient’s subjective experience of insomnia (assessed with the ISI) — did not improve meaningfully within the dosing window, remaining in the moderate range from d1 to d12. As discussed below, the substantial subjective improvement emerged only in the longer-term window, parallel to continued weight gain, and is therefore not attributable to leptin action during dosing alone.

The objective sleep architecture, by contrast, changed during dosing in a direction that points to deeper and more consolidated sleep (Fig. 3). Sleep depth, indexed by the proportion of slow-wave sleep (N3), increased (+ 7%), and nocturnal sleep continuity improved, with a marked reduction of wakefulness after sleep onset (WASO − 29%). The proportion of time asleep while in bed (sleep efficiency) remained stable. At the same time, total sleep duration (TST) was reduced (− 18%). Pre-treatment wakefulness after sleep onset had been elevated above age norms and moved into the normative range under treatment, while slow-wave sleep exceeded age norms both before and during treatment, increasing further. These changes occurred against a background of shorter time in bed (482 → 399 min) and earlier morning waking (lights-on 05:02 → 04:39), reflecting the persistent early-morning awakening present on both nights.

This combination — deeper, more continuous but shorter sleep — is consistent with experimental evidence from animal models that leptin promotes slow-wave sleep and stabilises sleep continuity through direct action on hypothalamic sleep-wake nuclei ([26]; [49]). It also aligns with the longitudinal data of Lindberg et al., [43], who found slow-wave sleep, BMI and leptin to be closely associated in AN, all three increasing under treatment without leptin administration, though without a causal interpretation [43]. In our case the increase in sleep depth occurred together with only a modest BMI increase and alongside a marked improvement in sleep continuity — a pattern compatible with a leptin-related contribution. Notably, [12] report that weight restoration alone does not consistently normalise total sleep time and wakefulness after sleep onset; in the present single case this is compatible with, but does not establish, a leptin-related contribution to the early change in sleep continuity over and above weight gain alone [12].

REM sleep did not increase in our case, in apparent contrast to rodent findings of increased REM after local leptin administration in the ventrolateral preoptic nuclei [49]. This is plausibly explained by the early-morning awakening that truncated the second half of the night, when REM is normally most prominent. Spiegel et al., [56] showed that experimental sleep restriction in healthy young men reduced 24-hour leptin levels (− 19%), maximal nocturnal leptin (− 2%) and diurnal leptin amplitude − 20%), in association with a signifcant decrease in slow-wave sleep [56]. If the early-morning awakening in our case is conceptualised as a form of sleep restriction, the persistent elevation of sleep depth under leptin replacement is compatible with this bidirectional sleep-leptin relationship. In the adult cohort with AN of Abdou et al., [1], slow-wave sleep was higher in healthy controls than in patients [1].

The actigraphic measures of motor activity and circadian organisation showed a parallel attenuation of the starved state (Fig. 4). Overall daytime activity level (the rhythm-adjusted mean, MESOR) and the amplitude of the daily activity rhythm both decreased (− 10% and − 16%). Elevated motor activity during chronic starvation— captured by exactly these parameters—has been described in mouse models of AN and decreases under refeeding, indicating attenuation of starvation-associated hyperactivity [64]. Because the animal data describe this reversal under refeeding without leptin administration, the early occurrence of the same pattern under metreleptin in our case raises the possibility of an additive or accelerating contribution of leptin signalling. This is consistent with rat data showing that continuous leptin administration prevents and suppresses semi-starvation-induced hyperactivity, and with reduced subjective motor restlessness in patients with AN with rising leptin levels [22].

The timing and robustness of the rhythm changed in the same direction. The timing of the daily activity peak (acrophase) shifted earlier (14:20 → 13:36), reversing the later activity peak that has been described during chronic starvation and that shifts back under refeeding [64]. The goodness of fit of the activity data to a 24-hour rhythm (rhythm percent) decreased (0.73 → 0.52) and the total amount of activity fell (− 15%), together indicating hat the daytime activity profile became less rigidly over-driven. The day-night contrast (relative amplitude) was largely preserved (0.965 → 0.924). The pre-treatment profile may therefore be best understood as a starvation-associated over-organisation of daytime activity—high amplitude and rhythmicity—that attenuated toward a less starvation-dominated pattern over the period of leptin administration and early weight gain.

### Longer-term window (d12 → d + 60)

The changes after the end of dosing unfolded during substantial further weight gain and without continued leptin administration. They are therefore temporally associated with both the preceding leptin exposure and continued weight gain, with weight gain as the more plausible dominant contributor; the two cannot be separated in this single case. The density of clinical documentation in this window was lower (no repeat polysomnography, Cosinor analysis or VAS), so this section is correspondingly briefer.

#### Weight and symptoms of AN

Across this window the patient gained a further 5.7 kg, reaching a BMI of 18.3 kg/m². The two starvation-linked dimensions that had improved during dosing — acute eating-disorder cognitions and behaviour and compulsive exercise—were essentially maintained at their new lower level, whereas the broader cognitive and personality-related features and depressive symptoms rose above the pre-treatment level (+ 7% and + 10%, respectively; Fig. 1). The temporal alignment of this rise with continued weight gain is striking: body- and shape-related fears, ineffectiveness and perfectionism are known to improve more slowly than overt behavioural symptoms and may transiently intensify during weight restoration [12, 43], and depressive symptoms in early refeeding can be exacerbated rather than alleviated [39].

The dissociation between these two sets of dimensions—early improvement of the starvation-linked symptoms under leptin, transient worsening of the broader cognitive-affective experience during further weight gain—is consistent with the proposal that leptin replacement primarily targets the symptom dimensions of starvation, whereas the personal experience of weight restoration is regulated by other processes that may temporarily intensify even as biology improves. We regard this as a clinically meaningful tension that may contribute to emotional destabilisation during further weight gain, and therefore as a strong argument for accompanying psychotherapy during and after metreleptin treatment in AN, irrespective of the impressive short-term biological response.

### Symptoms of sleep quality and sleep disorder

Perceived sleep quality (subjective insomnia, ISI) improved substantially only in this window, moving from the moderate into the subthreshold range (15 → 8; Fig. 3), driven primarily by better sleep maintenance and less early-morning awakening. The dissociation between objective sleep architecture, which improved during dosing, and subjective insomnia, which improved predominantly with continued weight gain afterwards, is one of the more striking findings of this case. It accords with reports that subjective sleep quality in AN tracks illness severity and quality of life independently of BMI and emotional symptoms [7, 20, 44] and with the observation that subjective sleep in patients with AN receiving metreleptin improves over time and tends to track higher BMI [4, 30, 45].

Among the objective measures, within-day activity fragmentation (IV) continued to decrease across both windows, in line with the reduced fragmentation reported for AN under refeeding [20, 41], while the day-night contrast (relative amplitude) was preserved and the transient rise in day-to-day stability (IS) during dosing — plausibly reflecting a temporarily more constrained inpatient routine — did not persist (Fig. 4). Together with the Cosinor changes, this resembles the reversal of starvation-induced circadian alterations described in animal models [64], with the qualification that here the changes occurred in temporal proximity to leptin administration. Disentangling leptin-specific from weight-related contributions would require animal studies administering leptin under conditions of stable body weight.

#### Conclusions and implications for future studies

Several conclusions emerge from this case when the data are organised along the two assessment windows.

First, the short-term course in this patient, temporally associated with metreleptin administration, was characterised by changes in symptom dimensions tightly linked to starvation biology: a marked reduction of acute eating-disorder cognitions and behaviour (dietary restriction, weight and shape concerns) and a decrease of compulsive-exercise features (already in the subclinical range); attenuation of starvation-associated motor hyperactivity, reflected in a lower overall daytime activity level and a flatter, earlier and less rigidly over-driven activity rhythm; and changes in sleep architecture, with deeper slow-wave sleep and better nocturnal sleep continuity. These short-term changes mirror findings from rodent studies of starvation reversal [22, 64] and from previous off-label metreleptin case reports in AN [4, 27, 30, 45], and add the first concurrent objective sleep evidence from polysomnography (in a human patient) to this picture.

Second, the longer-term changes after the end of dosing were temporally associated predominantly with ongoing weight gain rather than with concurrent leptin administration. The starvation-linked dimensions that had improved during dosing — acute eating-disorder cognitions and behaviour and compulsive exercise—were maintained, whereas the broader cognitive and personality-related features of AN (such as ineffectiveness, perfectionism and interoceptive disturbance) together with depressive symptoms transiently increased above pre-treatment levels. Perceived sleep quality, in the form of subjective insomnia, improved substantially only in this longer-term window, parallel to continued weight gain.

Third, the dissociation between symptom dimensions in Fig. 1 is, in our view, the most clinically relevant pattern of this case. It is compatible with the possibility that leptin replacement is associated with rapid attenuation of starvation-related symptom dimensions while the broader cognitive-affective experience of weight restoration may remain unchanged or even transiently intensify; this remains a hypothesis-generating observation. We propose that this configuration warrants consistent psychotherapeutic accompaniment during and after pharmacological treatment, so that any early changes during the period of leptin administration can be built upon over the longer term.

Several questions for future studies arise from these observations. First, are the changes in objective sleep architecture under leptin sustained over time or do they primarily reflect an acute effect during active dosing? PSG should therefore be repeated at follow-up in future studies. Second, what role do sleep depth (N3) and sleep continuity (WASO) play in weight rehabilitation and recovery in AN? Third, is the improvement in subjective sleep quality more closely associated with weight restoration than with leptin administration, as the present temporal pattern is compatible with, and does this improvement contribute to long-term clinical outcomes? Fourth, what role does the observed pattern of circadian changes—attenuation of a starvation-associated over-organisation of daytime activity, with preserved day-night contrast — play in the pathophysiology and recovery process of AN?

To inform future decisions on the indication for leptin treatment, deep phenotyping of AN—including objective measures of sleep, circadian rhythmicity, and motor activity—will be essential. In a randomised controlled trial setting for metreleptin in AN, pre-, post- and follow-up sleep assessments using both subjective and objective sleep measures should be integrated. MotionWatches are particularly valuable for sleep and activity monitoring in AN, especially given the increasing use of outpatient and telemedicine-based treatment approaches.

A key limitation of this case report is that the observed changes during treatment cannot be attributed to leptin alone. The treatment attempt took place in an inpatient setting, where the patient concurrently received treatment as usual, including structured refeeding and psychotherapeutic care. Because the case was managed in a structured inpatient environment, standardised ward routines (sleep–wake schedule, meal timing, light exposure and daytime activities) and the patient’s stable co-medication represent additional influences on sleep timing and architecture. Although the co-medication was left unchanged throughout the dosing window, cumulative pharmacological effects and the effects of concurrent refeeding cannot be separated from a possible leptin-related contribution. Any leptin-related contribution therefore cannot be disentangled from the effects of concurrent treatment and weight gain, and the findings should be regarded as hypothesis-generating rather than confirmatory. A further limitation is that the outcomes were assessed retrospectively and were not pre-specified endpoints. In particular, the actigraphy-based Cosinor and non-parametric circadian rhythm parameters were derived post hoc from recordings acquired during clinical routine rather than for a prospectively registered analysis plan. The absence of a priori hypotheses and formal correction for multiple comparisons increases the risk of selective reporting and observer bias, so the observed associations should be interpreted with caution and treated as descriptive and hypothesis-generating. These constraints are inherent to the single-case, real-world design and should be addressed in prospective, pre-registered studies with predefined primary endpoints.

## Data Availability

No datasets were generated or analysed during the current study.

## Abbreviations

AgRP: Agouti-related peptide (hypothalamic neurons)
AHI: Apnoea–hypopnoea index
AI: Artificial intelligence
AN: Anorexia nervosa
BDI-II: Beck Depression Inventory-II
BMI: Body mass index
CET: Compulsive Exercise Test
CHEDE-Q: Child Eating Disorder Examination Questionnaire
d1: Day 1 (pre, start of dosing)
d12: Day 12 (post, end of dosing)
d + 60: Day 60 after end of dosing (follow-up)
DSM-5: Diagnostic and Statistical Manual of Mental Disorders, 5th edition
DZKJ: Deutsches Zentrum für Kinder- und Jugendgesundheit (German Center for Child and Adolescent Health)
EDI-2: Eating Disorder Inventory-2
EEG: Electroencephalography
FDA: U.S. Food and Drug Administration
HR: Heart rate
ICD-10: International Classification of Diseases, 10th revision
IGF-1: Insulin-like growth factor 1
IS: Interdaily stability (NPCRA parameter)
ISI: Insomnia Severity Index
IV: Intra-daily variability (NPCRA parameter)
MESOR: Midline Estimating Statistic Of Rhythm (Cosinor parameter)
N1, N2, N3: Non-REM sleep stages 1, 2, 3
NPCRA: Non-parametric circadian rhythm analysis
NREM: Non-rapid eye movement (sleep)
POMC: Pro-opiomelanocortin (hypothalamic neurons)
PSG: Polysomnography / polysomnographic
RA: Relative amplitude (NPCRA parameter)
RCT: Randomised controlled trial
REM: Rapid eye movement (sleep)
SE: Sleep efficiency
SPT: Sleep period time
TIB: Time in bed
TSQM: Treatment Satisfaction Questionnaire for Medication
TST: Total sleep time
VAS: Visual analogue scale
WASO: Wake after sleep onset
